# Cardiac Autonomic Derangement Is Associated with Worse Neurological Outcome in the Very Early Phases of Ischemic Stroke

**DOI:** 10.3390/jcm8060852

**Published:** 2019-06-14

**Authors:** Eleonora Tobaldini, Roberto M. Sacco, Serena Serafino, Michele Tassi, Gianluca Gallone, Monica Solbiati, Giorgio Costantino, Nicola Montano, Giuseppe Torgano

**Affiliations:** 1 Department of Internal Medicine, Fondazione IRCCS Ca’ Granda, Ospedale Maggiore Policlinico, 20122 Milan, Italy; eleonora.tobaldini@unimi.it (E.T.); roberto.mr.sacco@gmail.com (R.M.S.); 2 Department of Clinical Sciences and Community Health, University of Milan, 20122 Milan, Italy; michele.tassi@studenti.unimi.it (M.T.); monica.solbiati@unimi.it (M.S.); Giorgio.costantino@unimi.it (G.C.); 3 Department of Anesthesia, Critical Care and Emergency, Fondazione IRCSS Ca’ Granda, Ospedale Maggiore Policlinico, 20122 Milan, Italy; serafinoserena@gmail.com (S.S.); gianluca.gallone@studenti.unimi.it (G.G.); giuseppe.torgano@gmail.com (G.T.)

**Keywords:** acute ischemic analysis stroke, autonomic, sympathetic, heart rate variability, spectral analysis, symbolic

## Abstract

Background: Acute ischemic stroke (AIS) is associated with autonomic dysfunction. We evaluated the prognostic value of heart rate variability (HRV) and the role of stroke localization and reperfusion treatment (RT) on autonomic control. Methods: Patients with AIS and sinus rhythm were enrolled in the emergency department. Autonomic parameters were recorded at the onset and after a potential RT. Neurological deficit was assessed using the National Institute of Health Stroke Scale (NIHSS) at the onset and residual disability with modified Rankin Scale (mRS) at 3 months. Two analyses were used to assess HRV. Low frequency (LF) and high frequency (HF) are, respectively, markers of sympathetic and respiratory vagal modulation in spectral analysis. Symbolic analysis provides pattern with no variation (0V%) as an index of sympathetic modulation and pattern with two like variations (2LV%) and pattern with two unlike variations (2UV%) as markers of vagal modulation. Results: We enrolled 41 patients. Twenty-seven underwent RT. A prevalent parasympathetic modulation was found in patients with NIHSS ≥14. The group with mRS 3–6 exhibited a higher 2UV% and lower 0V%. Right-sided strokes were associated with a higher respiratory vagal control. RT had no effects on HRV parameters. Conclusions: In the very early phases of AIS, a decreased 0V% and an increased 2UV% may reflect a loss of sympathetic oscillation, predicting a poorer 3 month-outcome.

## 1. Introduction

Acute ischemic stroke (AIS) is one of the major causes of disability and mortality worldwide and it is expected to increase in the next years, due to the aging of the population [1]. The prognosis of these patients shows a great inter-individual variability depending on the patient’s premorbid condition, age, stroke severity, and post-stroke complications [2]. The relationship between lesions of the central nervous system and heart dysfunction has been known [3]. Clinical and experimental evidence suggests that a disorder of cardiac autonomic control (CAC) can contribute to this relationship [4,5].

Preliminary data from literature showed that acute stroke could alter the cardiovascular autonomic control, with myocardial injuries, electrocardiogram (ECG) abnormalities, cardiac arrhythmias, and a poor outcome [5,6]. In fact, increased sympathetic modulation and a lower heart rate variability (HRV) correlate with a poor outcome after acute stroke [7]. In addition, the final clinical outcome of patients with AIS is determined by the interplay of several pathophysiological factors whose relative importance remains to be determined [5]. Although AIS can be associated with neurological causes of death, most of the deaths between the first week and the third months after the stroke occur for non-neurological causes [8].

Heart rate variability is a non-invasive and reliable tool to investigate the autonomic nervous system control of cardiovascular functions [9]. Several studies have shown an altered HRV in patients with AIS compared to controls [10,11,12] and few studies investigated the prognostic effects of HRV parameters in AIS patients, although with contrasting results [13,14,15,16]. Another challenging question concerns the possible implication of specific ischemic areas in this process.

The hypothesis of a “laterality” in autonomic control was first argued thanks to experimental strokes in animals [17]. Right hemisphere lesions seem to be responsible for an altered sympathetic modulation [18,19], with a possible primary role of the insular cortex [20].

Thus, our study aimed to evaluate the prognostic role of HRV parameters on neurological outcomes, using both linear and non-linear approaches for the analysis of autonomic control, taking into account the site of the lesion.

## 2. Materials and Methods

### 2.1. Experimental Protocol

The experimental protocol was approved by the Ethical Committee of Fondazione IRCCS Ca’ Granda, Ospedale Maggiore Policlinico, Milan, Italy (approval number 332_2016bis, approved on 27/06/2016). All the patients signed an informed consent. From September 2015 to March 2018, we enrolled 41 patients admitted to the Emergency Department, Fondazione IRCCS Ca’ Granda, Ospedale Maggiore Policlinico (Milan, Italy) with a diagnosis of acute ischemic stroke. Exclusion criteria were primary intracerebral hemorrhage, absence of stable spontaneous sinus rhythm on the ECG at presentation, clinically relevant pre-existing neurological deficit; generalized seizures at the onset of stroke symptoms; severe organ failure; cerebral or extracerebral cancer disease with reduced life-expectancy; mechanical ventilation, consent refusal.

Patients were evaluated at the time of presentation in the emergency department (T0), immediately after any reperfusion therapy (T1), 7 days (T7) and 3 months after the acute event. At T0, patients underwent the assessment of autonomic control by the recording of ECG and respiration signal through a thoracic piezoelectric belt (sampling rate 1000 Hz) with an ad hoc telemetric system device (BT16, FM, Monza, Italy) for 10 minutes.

For the analysis of cardiac autonomic control, we applied two different complementary tools, spectral and symbolic analysis of HRV on samples of 250 ± 50 beats associated with stable breathing.

Clinical data were collected and included: age, gender, active smoking, comorbidities (hypercholesterolemia, history of TIA or stroke, hypertension, diabetes mellitus, heart failure, atrial fibrillation or flutter -not present during ECG recording with BT16) and current therapy (especially if taking beta-blockers).

At T0, stroke severity was quantified by the National Institutes of Health Stroke Scale (NIHSS): an 11-items scale evaluating specific skills. The overall score ranges from 0 to 42 with higher scores indicating more severe neurological impairment. We considered NIHSS score at the onset as a short-term outcome, assuming an NIHSS score ≥14 as an indicator of worse outcome [21]. The modified Rankin Scale (mRS) is a common scale which measures the degree of dependence in daily activities of patients affected by stroke [22]. This scale is divided into 6 degrees: 0 is assigned to a patient without symptoms, 5 to a patient with severe disability and requiring constant care, and 6 in the case of death. We considered mRS at 3 months as a medium-term outcome. Dichotomous cut-off of 0 to 2 versus 3 to 6 were used in order to categorize patients in two disability groups [23].

Reperfusion therapy is the specific treatment for AIS and it is time-dependent. It aims to restore blood flow and save the penumbral zone from necrosis, either by intravenous (IV) thrombolytic drugs (recombinant tissue plasminogen activator or r-tPA) or by endovascular treatment (intra-arterial tPA and/or mechanical thrombectomy). To avoid hemorrhagic transformation of AIS or other bleeds as a side effect, reperfusion therapy must be reserved for patients who respect inclusion and exclusion criteria; for the others, secondary prevention strategies should be provided [24]. In this study, IV thrombolytic therapy (r-tPA at 0.9 mg/kg; 90 mg as maximum total dose; 10% as IV bolus and the remainder infused over 60 minutes) was planned for patients older than 18 years; affected by an AIS with symptom onset within 4.5 hours; without a history, a high suspicion or a certainty of cerebral hemorrhage; without a current or recent severe bleeding; without a history of bleeding disorder or administration of IV heparin in the previous 48 hours and a prolonged aPTT. Other relative contraindications were also considered [25,26,27]. Endovascular treatment was planned for AIS patients with large-vessel occlusion who received intravenous r-tPA at admission if allowed, with symptoms onset within 6 hours or in case of contraindications to IV r-tPA [8,25,26,28].

### 2.2. Data Analysis

#### 2.2.1. Spectral Analysis

Spectral analysis (SpA) of HRV is a computed method that uses an autoregressive model to identify the main oscillatory components embedded in the heart period and blood pressure time series. Namely, the SpA identifies three main rhythmic components: (a) very low frequency (VLF), frequency band bounded between 0.003–0.04 Hz, marker of humoral and hormonal activity; (b) low frequency component (LF), frequency band bounded between 0.04–0.15 Hz, marker of sympathetic modulation, and (c) high frequency component (HF), frequency band between 0.15–0.4 Hz, marker of vagal modulation and synchronous with respiration.

LF and HF oscillations are expressed both in absolute units (ms^2^) and in normalized units (nu). Normalized units represent the relative amount of each component with respect to the total power. The algorithm also calculates the LF/HF, the ratio between LF and HF, which is considered an index of the “sympatho-vagal balance.”

The power spectrum of respiration contains a unique component, HF. In physiological conditions, its central frequency is very close to the heart rate HF component [9].

#### 2.2.2. Symbolic Analysis

Recently, a new non-linear method, symbolic analysis (SA), has been validated as a reliable tool to detect non-reciprocal changes of sympathetic and parasympathetic modulation in healthy and diseased patients [29]. SA is based on the transformation of time series into a sequence of symbols, the construction of patterns, the reduction of patterns into four families, and the evaluation of their occurrence. Thus, we can identify four families: 0V%, pattern with no variation, 1V %, pattern with one variation, 2LV%, patterns with two like variations, and 2UV% pattern with two unlike variations. It has been demonstrated that 0V% is a marker of sympathetic modulation while 2LV% and 2UV% are markers of vagal modulation [29,30,31].

### 2.3. Statistical Analysis

Data were analyzed using SigmaStat software (2016 Systat Software, Inc., Chicago, IL, USA). Results were expressed as the median and interquartile range (25–75° IQR). A normality test (Shapiro–Wilk) was applied to all collected values. When a non-normal distribution was found, group differences were examined by Mann–Whitney two-sample test. A *p* value < 0.05 was considered statistically significant.

## 3. Results

We recruited 41 patients (28 men and 13 women). Clinical and demographical data of the patients are reported in Table 1.

Mean age was 68 ± 12.8 years. A previous stroke or transient ischemic attack was found in 36.6% of the total sample. Main stroke risk factors were hypertension (68.3%), diabetes (22%), heart failure (7.3%), and hypercholesterolemia (51.2%). Past medical history of atrial fibrillation (AF) or flutter was reported in 3 patients, 12 patients already had beta-blockers in their home-therapy.

As to neurological assessment, mean NIHSS at T0 was 9.8 ± 6.4 and 29 patients had an NIHSS <14 and 12 had an NIHSS ≥14.

The modified Rankin scale was dichotomized in two ranges of different disability: mRS 0–2 (16 mRS 0, 5 mRS 1, 5 mRS 2, total 26 patients) and mRS 3–6 (3 mRS 3, 2 mRS 4, 3 mRS 5, 6 mRS 6, total 18 patients). A patient was lost at third-month follow-up. An mRS of 6 corresponds to death: 6 patients (15%) died within three months after the ischemic event. As to acute treatment, 27 patients (65.8%) underwent RT: 18 received intravenous rtPA, 6 a combination of IV rtPA and thrombectomy, 2 only thrombectomy and the last one thrombectomy associated to intra-arterial rtPA. At T1, it was possible to record data for only 19 patients out of 27 for technical reasons. As to T7, we were able to record 15 patients out of 27 (due to different lengths of hospitalization).

Our primary outcome was to evaluate the prognostic role of autonomic parameters in ischemic strokes. Comparing patients with NIHSS <14 and NIHSS ≥14, we found a greater 2UV% in the group with a more severe stroke at the onset (12.6 (8.8–20.2) vs. 20.7 (13–32.2), *p* = 0.04). No statistically significant results emerged from the spectral analysis data (See Table 2).

**Figure 1:**
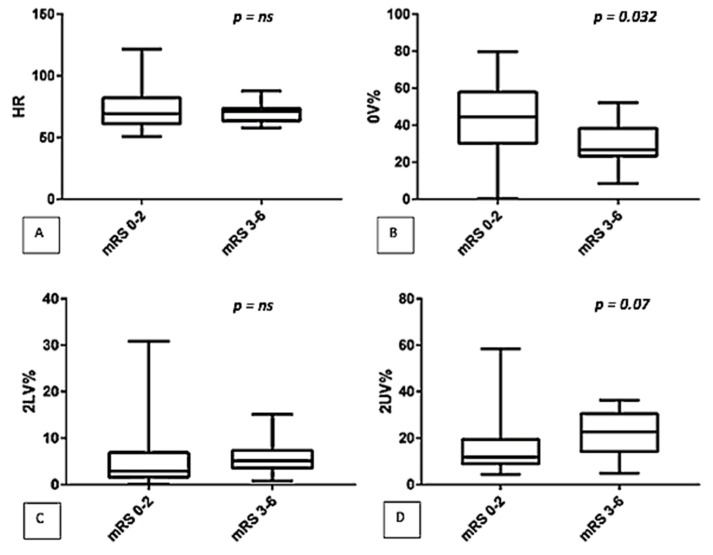
Comparison of autonomic parameters (symbolic analysis: (**A**) HR, (**B**) 0V%, (**C**) 2LV%, (**D**) 2UV%) between two classes of disability (mRS 0–2 vs. mRS 3–6).

After three months, patients were classified according to their mRS. The comparison between the two groups (mRS 0–2 vs. mRS 3–6), showed that patients with worse outcome (mRS 3–6) had a lower 0V% (44.5 (29.6–58.4) vs. 26.6 (22.7–39), *p* = 0.032) and a higher 2UV% (11.8 (8.4–20) vs. 22.5 (13.6–31), *p* = 0.007) (See Figure 1). No significant differences were detected by spectral analysis (LF/HF: 2.1 (1–7.3) vs. 2 (0.7–5.8), *p* = ns).

As a secondary outcome, we examined the possible role of stroke localization on the autonomic derangement. According to our data, right hemispheric lesions (*n* = 21) presented a greater vagal modulation compared to left ones (*n* = 19), as shown by a greater 2LV% (5.7 (2.7–9.7) vs. 2.9 (1.2–4.4), *p* = 0.022; see Table 3). One patient had a bilateral stroke; therefore, he was not included in this analysis. Comparisons between anterior and posterior lesions did not disclose any significant difference, as well as comparing insular to non-insular lesions (See Table 3).

Finally, we evaluated the effects of reperfusion therapy on autonomic control (T0 vs. T1), but we did not find any significant results. Similarly, comparing T0 to T7, there were no significant changes in autonomic parameters (see Table 4).

**Figure 2:**
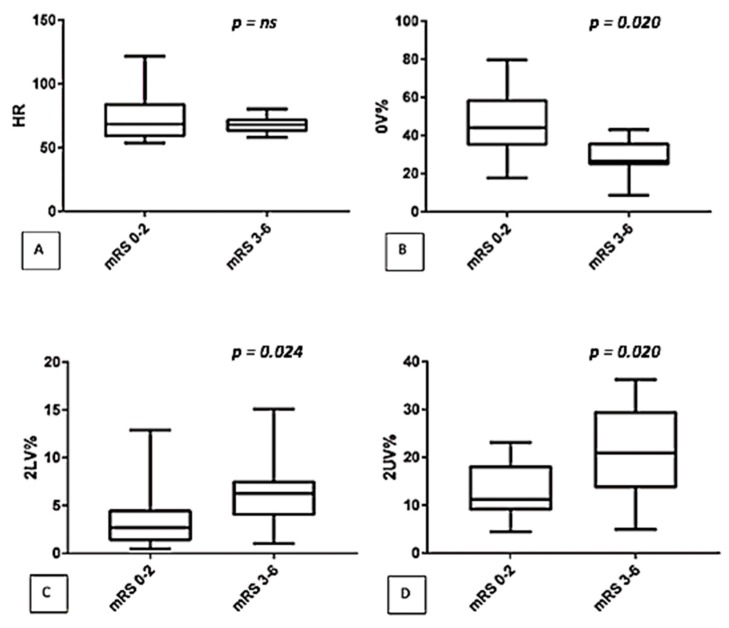
Comparison of autonomic parameters (symbolic analysis: (**A**) HR, (**B**) 0V%, (**C**) 2LV%, (**D**) 2UV%) between two classes of disability (mRS 0–2 versus mRS 3–6) in the subgroup of treated patients.

In addition, we correlated autonomic control with mRS in the subgroup of 27 treated patients at the onset (T0) and we found results superimposable to those found in the main population: in fact, a higher 2LV% and 2UV% and a lower 0V% were observed in patients with the most severe outcome at the third month follow-up (See Figure 2).

## 4. Discussion

This study shows for the first time that a specific autonomic pattern in the very early phases of ischemic stroke is associated with the worse outcome.

The main findings of the present study are the following: patients with a more severe stroke at admission are characterized by a predominant vagal modulation; patients with the worse clinical outcome at 3 months exhibit a loss of sympathetic rhythmic modulation and a persistent vagal modulation; patients with right hemispheric lesions present a greater vagal modulation compared to left ones, while no differences of autonomic patterns are found comparing anterior with posterior strokes and insular with non-insular strokes; no acute effects of reperfusion therapy are observed on autonomic control; finally, symbolic analysis has been a more reliable tool than power spectral analysis in detecting changes of autonomic oscillations in this population.

An autonomic derangement in patients with AIS was already observed in other studies [10,11,12,32]. This phenomenon may rely on an unbalanced activity of sympathetic and vagal branches. According to our results, the degree of severity in the very early phases of stroke is correlated to a predominant vagal modulation, as shown by a greater 2UV% in patients with NIHSS ≥14. Thus, we can suppose a persistent vagal respiratory control in these patients, with a loss of sympathetic modulation. The same autonomic pattern seems to be associated to a worse midterm disability: in fact, we found a prevalent vagal modulation combined to a greater clinical impairment (mRS) three months after the event, with a higher 2UV% and a reduced 0V% in this group (mRS 3–6). These results seem in line with those found by Graff et al., who reported that a greater HF and a lower LF/HF were associated with a worse 90-days outcome in patients with ischemic lesions [33]. These results suggest that vagus is over-modulated by respiratory inputs while the sympathetic branch is characterized by a loss of rhythmical oscillatory pattern. A similar autonomic profile represents a stiff system, which is less capable to adequately respond to external and internal stressors stimuli. In other acute clinical conditions, like community-acquired pneumonia [34], it has been shown that such an autonomic profile is associated with worse clinical conditions.

The role of stroke localization is a challenging issue. Previous authors already examined the possible implication of right or left hemisphere in the autonomic changes [5,10,18,19,35,36]. Right-sided ischemic lesions were also studied in experimental settings by occluding the middle cerebral artery in rats and cats [20]. They appear to be associated with more autonomic and cardiac disorders compared to the left ones. Insular lobes in both experimental and clinical studies seem to hold a predominant role [5,13,16,20,37]. According to our data, we can highlight a greater vagal respiratory control in patients with right lesions compared to left-sided strokes, as shown by a higher 2LV%. This result needs to be confirmed on a larger number of patients, in order to verify specific changes of autonomic parameters according to the site of the lesion. We also performed an additional comparison between anterior and posterior lesions, but we did not find any significant difference in autonomic parameters. Neither insular lesions, regardless of the side, showed a relevant role among our patients.

Regarding insula localization, our result seems to be in contrast with previous findings [5,13,16,20,37]. However, we can hypothesize that these results are affected not only by the small sample of patients but also by the fact that recordings have been done in the very early phases of stroke. This timing could not be able to detect different autonomic profiles according to the site of lesions.

Reperfusion treatments are the cornerstone of stroke management in the ED [24]. Whenever possible, they can change patients’ clinical histories. We found no differences comparing patients’ autonomic parameters before and after the event, either considering recordings performed a week later.

We also compared autonomic profile between the two disability groups (mRS 0–2 and 3–6) at T0 in the treated patients, and we found that similarly to our main population, a worse outcome is associated with a predominant vagal and a loss of sympathetic modulation. Thus, we can infer that autonomic derangement may not be affected by treatment. However, we have no medical literature about this topic and this a very preliminary result, which has to be tested to a larger and more uniform population.

It is well known that the response to available therapies is not homogeneous; thus, we could speculate that these different responses could be influenced by specific autonomic patterns. On the other hand, it is possible that by modifying cardiac autonomic control in acute phases of ischemic stroke, we would be able to improve the clinical responses to different treatments. In addition, these results seem to suggest that specific cardiac autonomic profiles may be helpful for the stratification of high-risk patients that could benefit more intensive treatments. Future ad hoc studies are warranted in order to shed new lights on this population.

In our study, we performed two different kinds of analyses, i.e., a linear and a non–linear approach. No changes in autonomic modulation was found by classical spectral analysis; however, the symbolic analysis was more reliable in detecting changes of autonomic control in our population, as already shown in other studies [29,34,38]. Since a linear approach is based on the concept of “sympatho-vagal balance,” we can suppose that a non-linear analysis may be able to detect non-specular changes in the activity of the two branches, especially in acute clinical settings, characterized by the co-activation of several biological systems.

### Limitations

The small sample size is the most important limit of this study. Due to the acute setting and the difficult management of prompt therapy, it was not always possible to collect data. Another issue is the high number of drops out, determined by technical and logistic reasons. Second, the analysis of autonomic control is narrowed to patients in sinus rhythm, excluding many cardio-embolic strokes with AF detected at the admission. Thus, our results are representative of a portion of the entire population with a stroke. Finally, we did not record sympathetic activity, as muscle sympathetic activity, which could have added complementary information on autonomic control.

## 5. Conclusions

Ischemic stroke patients with worse early (NIHSS at admission) and mid-term (mRS at 3 months) outcome seem to share an autonomic pattern characterized by a predominant vagal modulation and the loss of rhythmic sympathetic activity. This phenomenon may reflect a greater cardiovascular and arrhythmic risk due to an ischemic lesion. If our data are confirmed by future studies, we wish for risk stratification of patients with ischemic stroke and consequent preventive and therapeutic approaches targeting autonomic damage.

## Figures and Tables

**Table 1:** Demographic and clinical data of the main population.

Demographic and Clinical Data	Male (28)	Female (13)	All (41)
Age (years)	67.8 ± 12.6	68.4 ± 13.2	68.0 ± 12.8
Active smoking	10 (35.7)	2 (15.4)	12 (29.3)
Hypercholesterolemia	14 (50)	7 (53.8)	21 (51.2)
History of transient ischemic attack or stroke	8 (28.6)	7 (53.8)	15 (36.6)
Hypertension	19 (67.9)	9 (69.2)	28 (68.3)
Diabetes mellitus	8 (28.6)	1 (7.7)	9 (22)
History of atrial fibrillation or flutter	1 (3.6)	2 (15.4)	3 (7.3)
Heart failure	2 (7.1)	1 (7.7)	3 (7.3)
Beta-blockers medication	9 (32.1)	3 (23.1)	12 (29.3)
Systolic blood pressure	156.8 ± 25.7	145.4 ± 23	153.4 ± 25.2
Diastolic blood pressure	89.8 ± 16.5	81.2 ± 19.8	87.3 ± 17.7

Results are presented as mean ± standard deviation or number (%).

**Table 2:** Autonomic parameters according to the National Institute of Health Stroke Scale (NIHSS) at the onset of an acute event.

Autonomic Parameters	NIHSS <14 (29)	NIHSS ≥14 (12)	*p*
HR (bpm)	72.5 (61.7–83.7)	64.4 (59.5–71.9)	ns
Total Power (ms^2^)	612.8 (301.6–1334.6)	804.3 (308.8–1045.6)	ns
LF (n.u.)	56.7 (38.4–83.2)	49.6 (29.4–81.8)	ns
HF (n.u.)	25 (10.1–49.2)	27 (15.6–45.2)	ns
LF/HF	1.9 (0.7–7.6)	2.1 (0.8–4.7)	ns
0V%	38.9 (22.7–51.8)	26.2 (22.9–50.5)	ns
1V%	42 (32.8–48.6)	39.1 (34.3–46.6)	ns
2LV%	3.3 (1.3–8.5)	5 (2.7–7.1)	ns
2UV%	12.6 (8.8–20.2)	20.7 (13–32.2) *	0.04

Results are presented as median and interquartile ranges (25–75°). * *p* < 0.05 vs. NIHSS <14. HR: heart rate; LF: low frequency in normalized units; HF: high frequency in normalized units; LF/HF: ratio between LF and HF; 0V%: pattern with no variation; 1V%: pattern with one variation; 2LV: pattern with two like variations; 2UV%: pattern with two unlike variations.

**Table 3:** Autonomic parameters according to stoke localization.

Autonomic Parameters	Right Hemispheric Strokes (21)	Left Hemispheric Strokes (19)	*p*	Anterior Ischemic Lesions (33)	Posterior Ischemic Lesions (8)	*p*	Insular Ischemic Lesions (13)	Non-Insular Ischemic Lesions (28)	*p*
HR (bpm)	68.2 (61.7–77.8)	71.8 (60.2–84.4)	ns	71.8 (62.1–83.7)	61.2 (56.2–72.4)	ns	68.6 (62.3–86.5)	70.5 (58.5–79.7)	ns
Total Power (ms^2^)	941 (274.9–1720.4)	585.9 (272.9–940.8)	ns	612.8 (260–1334.6)	863.4 (540.5–976)	ns	896.2 (260–1632.8)	599.4 (333.2–1213.9)	ns
LF (n.u.)	53.1 (39–80.7)	56.7 (27.6–82.7)	ns	53.1 (30.1–83.3)	61.7 (33.1–80.4)	ns	58.1 (28.1–86)	54.9 (33.1–81.3)	ns
HF (n.u.)	27.7 (15.1–50.2)	26.7 (9.4–46.6)	ns	26.7 (10.1–47.6)	23.9 (12–52.2)	ns	27.3 (10.2–53.1)	25.8 (11.3–45.3)	ns
LF/HF	1.8 (0.8–4.4)	2.1 (0.7–8.7)	ns	1.9 (0.8–7.6)	2.1 (0.9–6.4)	ns	2.1 (0.8–8.5)	2 (0.8–6.4)	ns
0V%	31.9 (20.8–44.4)	44.1 (25.5–53.4)	ns	37.7 (21.7–49.6)	35.8 (25.2–51.3)	ns	37.3 (21.4–43.3)	37.1 (23.1–55.8)	ns
1V%	42.7 (34–47.9)	39.9 (33.1–49)	ns	42 (33.6–47.2)	43.9 (34.8–48.3)	ns	42 (35.3–47.5)	42.1 (30.8–48.2)	ns
2LV%	5.7 (2.7–9.7)	2.9 (1.2–4.4) *	0.022	4 (2.2–7.8)	3.4 (1.2–6.7)	ns	4.5 (1.2–7.8)	3.5 (2.1–7.3)	ns
2UV%	16.5 (9.8–23.4)	12.6 (9.4–20.1)	ns	15.1 (9.2–22.5)	12.7 (11.4–19.6)	ns	18.2 (10.9–24.9)	12.7 (8.9–20.3)	ns

Results are presented as the median and interquartile range (25-75°). * *p* < 0.05 vs. right hemispheric strokes. HR: heart rate; LF: low frequency in normalized units; HF: high frequency in normalized units; LF/HF: ratio between LF and HF; 0V%: pattern with no variation; 1V%: pattern with one variation; 2LV: pattern with two like variations; 2UV%: pattern with two unlike variations.

**Table 4:** Autonomic parameters before and after treatment.

Autonomic Parameters	T0 (19)	T1 (19)	*p*	T0 (15)	T7 (15)	*p*
HR (bpm)	66 (58.8–82.5)	65.8 (61.9–79.6)	ns	73 (57.9–84.4)	67.5 (57.5–74.4)	ns
Total Power (ms^2^)	712.4 (439.8–1266.6)	606.1 (247.6–1124.9)	ns	585.9 (434.9–1227.6)	400.9 (235.1–681.2)	ns
LF (n.u.)	42.5 (28.6–82.7)	50.9 (27.4–71.7)	ns	46.9 (31.7–82.7)	29.9 (13.5–64.2)	ns
HF (n.u.)	25 (10.9–51.9)	18.9 (13.3–33.5)	ns	15.3 (9.4–51.9)	30.9 (15.1–55.9)	ns
LF/HF	1.8 (0.7–6.8)	2.5 (0.8–6.2)	ns	3.1 (0.7–8.8)	1 (0.5–2.5)	ns
0V%	37.3 (26.3–45.7)	34.8 (22.3–48.8)	ns	37.7 (33–53.4)	36.5 (26.1–49.5)	ns
1V%	44.6 (35–46.3)	42.3 (36.3–46)	ns	45.2 (33.1–49)	42.5 (32.4–47.1)	ns
2LV%	3 (1.2–5.7)	3.6 (1.3–6.1)	ns	2.9 (1.2–5.7)	2.8 (1.3–6)	ns
2UV%	12.8 (9.5–20.5)	18.1 (11–26.1)	ns	12.3 (9–20.1)	17.7 (13–26.9)	ns

Results are presented as median and interquartile ranges (25°–75°). HR: heart rate; LF: low frequency in normalized units; HF: high frequency in normalized units; LF/HF: ratio between LF and HF; 0V%: pattern with no variation; 1V%: pattern with one variation; 2LV: pattern with two like variations; 2UV%: pattern with two unlike variations.
